# *LINC00313* regulates the metastasis of testicular germ cell tumors through epithelial-mesenchyme transition and immune pathways

**DOI:** 10.1080/21655979.2022.2073128

**Published:** 2022-05-16

**Authors:** Zhizhong Liu, Bairong Fang, Jian Cao, Qianyin Zhou, Fang Zhu, Liqing Fan, Lei Xue, Chuan Huang, Hao Bo

**Affiliations:** a Department of Urology, Hunan Cancer Hospital, the Affiliated Cancer Hospital of Xiangya School of Medicine, Central South University, Changsha, Hunan, China; b NHC Key Laboratory of Human Stem Cell and Reproductive Engineering, Institute of Reproductive and Stem Cell Engineering, School of Basic Medical Science, Central South University, Changsha, Hunan, China; c Department of Plastic and Aesthetic (Burn) Surgery, the Second Xiangya Hospital, Central South University, Hunan, Changsha, China; d Clinical Research Center for Reproduction and Genetics in Hunan Province, Reproductive and Genetic Hospital of CITIC-Xiangya, Changsha, Hunan, China; e Department of Pathology, Hunan Cancer Hospital, the Affiliated Cancer Hospital of Xiangya School of Medicine, Central South University, Changsha, Hunan, China

**Keywords:** Testicular germ cell tumor, TGCT, LINC00313, migration, invasion, immune

## Abstract

Testicular germ cell tumor (TGCT) is a relatively rare entity tumor, accounting for only 1% of all male cancers. However, it is the most common solid tumor in young men between 15 and 34 years old. Long noncoding RNAs (lncRNAs) are involved in various physiological and pathological processes. However, the functions of lncRNAs in TGCT have only rarely been investigated. LncRNAs associated with TGCT were identified using Gene Expression Omnibus (GEO) database and UCSC XENA database data mining. The effects of *LINC00313* on NCCIT cell migration and invasion were evaluated in transwell assays. The expression levels of epithelial-mesenchyme transition (EMT)-related proteins in cells knockdown of *LINC00313* were analyzed by Western blot. Correlation analyses between lncRNA *LINC00313* expression and copy number variation (CNV) and immune cell infiltration were carried out using The Cancer Genome Atl as (TCGA) data. The effect of Panobinostatin targeting *LINC00313* in TGCT cells was investigated. We observed higher *LINC00313* expression in TGCT. The migratory and invasive properties of TGCT cells were augmented by *LINC00313*, likely via its effects on modulating the expression of epithelial-mesenchyme transition (EMT) related proteins: CTNNB1, ZEB1, CDH2, Snail and VIM. Moreover, *LINC00313* expression and CNV correlated negatively with the infiltration of immune cells. In addition, Panobinostat might be a possible candidate drug to target *LINC00313* in TGCT. *LINC00313* performs important pro-migration and invasion functions in the pathogenesis of TGCT. *LINC00313* could be used as diagnostic, prognostic, immune marker and therapeutic target to develop effective treatment of TGCT.

## Graphical Abstract

**Figure:**
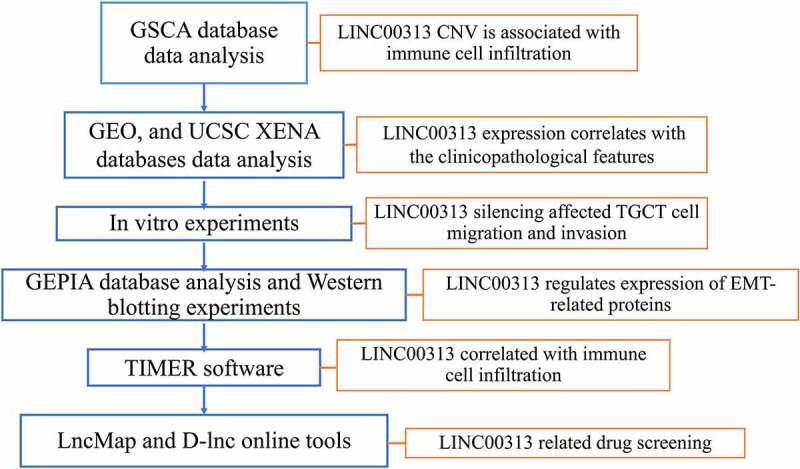

## Highlights

- LINC00313 could be a prognostic marker of TGCT.
- LINC00313 promote the migration and invasion of TGCT.
- LINC00313 influence the EMT signaling of TGCT.

## Introduction

Testicular germ cell tumor (TGCT) is a relatively rare malignancy, representing only 1% of all cancers in men. However, it is the most common solid tumor in young men between the ages of 15 and 34 [1]. TGCTs are classified broadly into seminomas, which resemble primordial germ cells (PGCs), and non-seminomas, which are either undifferentiated (embryonic carcinoma) or differentiated (teratoma, yolk sac tumor, choriocarcinomas) [2]. The incidence of TGCTs has increased worldwide during recent years, particularly in men of European descent, although a decline in mortality rates has been reported in western countries. For the clinical management of TGCTs, increased levels of lactate dehydrogenase, alpha-fetoprotein, and human chorionic gonadotropin are essential tools for diagnosis, risk assessment, and patient prognosis [3]. However, serum levels of these tumor markers are shown increased in only 60% of patients with TGCTs [4]. Hence, alternative and more functional markers should be investigated and introduced for the diagnosis and prognosis of TGCTs.

Noncoding DNA covers 95% of DNA sequences in the human genome, most of which are transcribed into various non-coding RNAs (ncRNAs). Testes express large numbers of ncRNAs, mainly microRNAs (miRNAs), piwi-interacting RNAs (piRNAs) and long noncoding (lncRNAs), which play roles in the regulation of gene expression. LncRNAs are conventionally defined as transcripts with lengths exceeding 200 nucleotides that are not translated into protein. Functions of lncRNAs rely on their capacity to bind and regulate a molecular partner, either via base-pair interactions or through their secondary structure [5]. Alterations in lncRNA expression might be involve in testicular germ cell tumorigenesis. This hypothesis is supported by the observation that many TGCT risk loci identified in a genome-wide association study (GWAS) are in the non-coding regions of the genome [6]. *PRY4-IT1*, a lncRNA produced within the intronic region of *SPRY4* (encoding sprouty RTK signaling antagonist 4), acts as an oncogene in TGCT development and its expression is silenced in TGCT cells [7]. Previously, we found that the expression of lncRNA *LINC00467* correlated positively with poor prognosis and the pathological grade of TGCT [8]. However, lncRNAs in TGCT have only been investigated rarely. Recently, some studies reported that lncRNA *LINC00313* promote tumorigenesis and metastasis in Human cancer. In this study, we explore the role of *LINC00313* in TGCT progression to understand the importance of the *LINC00313* in TCGT and provide insights into the role of *LINC00313* in the progression of TGCT.

To the best of our knowledge, the relationship between *LINC00313* and TGCT has not been previously reported. In this study, we firstly reported that *LINC00313* was overexpressed in TGCT. Therefore, this study aims to address this research question with the hope of discovering an additional therapeutic target and molecular marker of clinical significance in TGCT treatment and diagnosis.

## Materials and methods

### Online database data analysis

The Gene Set Cancer Analysis (GSCA; http://bioinfo.life.hust.edu.cn/GSCA/#/) database is an interactive web version tool developed by the research team of Huazhong University of Science and Technology [9]. It was used to analyze the relationship between *LINC00313* CNV and the infiltration of two immune cell types, CD8 + T cells and dendritic cells (DCs), in TGCT. The expression data of LINC00313 in normal samples, seminoma samples and non-seminoma samples are obtained from the GEO database (https://www.ncbi.nlm.nih.gov/geo/). These data are from the same dataset as GSE3218 [10,11]. Gene Expression Profiling Interactive Analysis (GEPIA, http://gepia.cancer-pku.cn/) was developed based on the TCGA and Genotype-Tissue Expression (GTEx) databases [12]. We used the UCSC XENA (https://xena.ucsc.edu/) online tool [13] to analyze the expression of *LINC00313* and its correlation with the survival of patients with TGCT from TCGA. TGCT patients were divided into two groups according to the expression of a certain gene (high or low expression). In addition, the GEPIA database was used to analyze the correlation between *LINC00313* and the expression of epithelial-mesenchymal transition (EMT) related genes, such as VIM (encoding vimentin, *ZEB1* (encoding zinc finger E-box binding homeobox 1), *SNAIL* (encoding snail family transcriptional repressor 1), *CTNNB1* (encoding catenin beta 1), and *CDH2* (encoding N-cadherin). We have analyzed interacted miRNAs associated with the lncRNA and genes by using Targetscan (https://www.targetscan.org/vert_72/) and miRcode (http://mircode.org/index.php)[14–16]. A model of EMT-related genes regulated by LINC00313 based on the prognostic classification of TGCT was constructed using the online tool ASSISTANT for Clinical Bioinformatics (https://www.aclbi.com/static/index.html#/) along with the R software package glmnet (v 4.1–1) and timeROC (v 0.4) based on TCGA TGCT cohort data. Sangerbox tools (http://sangerbox.com/ Index) were used to assess the relationships between LINC00313 expression levels and the immune score, stromal score, and immune cell enrichment score in TGCT. *LINC00313* involvement in immune cell and immune-related pathway was analyzed using ImmuLnc [17] tool (http://bio-bigdata. hrbmu.edu.cn/ImmLnc/index.jsp). Finally, LncMap [18] tool was used to analyze the relationship between *LINC00313* expression and the half-maximal inhibitory concentration (IC50) of various drugs.

## Cell culture and siRNA transfection

The human TGCT cell line, NCCIT, used in this study, was donated by the Associate Researcher Su-Ren Chen. NCCIT cells were cultured in Roswell Park Memorial Institute (RPMI)-1640 medium (Gibco, Grand Island, NY, USA) containing 10% fetal bovine serum (FBS; Gibco) at 37°C in 95% air and 5% carbon dioxide (CO_2_). Confluent cells were digested and passaged with 0.1% trypsin (Gibco). Negative control small interfering RNA (siRNA) and siRNAs targeting *LINC00313* were designed and synthesized by Tsingke Biotechnology Co., Ltd (Beijing, China). TGCT cells were prepared before transfection. Plasmid transfection was performed using Lipofectamine 2000 transfection reagent (Invitrogen, Waltham, MA, USA) according to the manufacturer’s instructions. All siRNAs were designed and synthesized by Ribobio (Guangzhou, China). LINC00313 siRNA-1: GCTTCCTGGATTGCATAAA; LINC00313 siRNA-2: GGCTCCTTCTCCTTACATA. Quantitative real-time reverse transcription PCR (qRT-PCR) with relative quantification was used to assess the knockdown efficiency of the siRNAs to identify the most effective molecule.

## Transwell cell migration and invasion experiments

Transwell migration and invasion assays were performed using 8.0 μm Transwell Permeable Supports (Corning Inc., Corning, NY, USA). Cells at a density of 5 × 10^4^ cells per well in 100 µl serum-free medium were seeded into the upper chamber pre-coated with Matrigel Matrix (BD Biosciences, San Jose, CA, USA), Then, 600 μl medium containing 15% FBS was added to the lower chamber. The time for the migration assay was 12 h and that for the invasion assay was 36 h, cells that did not invade through the membrane were mechanically removed with a cotton swab. Next, 4% paraformaldehyde was used to fix the cells on the bottom surface of the membrane for 10 min, which were then stained with crystal violet solution. The number of invaded cells was counted in five randomly selected fields.

## Western blotting

The cellular protein precipitates were extracted using radioimmunoprecipitation assay (RIPA) lysis buffer (Beyotime, Nanjing, China). Proteins were separated on 10% sodium dodecyl sulfate-polyacrylamide gel electrophoresis (SDS-PAGE) gels, electro-blotted onto a polyvinylidene difluoride (PVDF) membrane, and incubated with anti-VIM antibodies (Cell Signaling Technology, Danvers, MA, USA), anti-ZEB1 antibodies (Cell Signaling Technology, Danvers, MA, USA), anti-SNAIL antibodies (Cell Signaling Technology, Danvers, MA, USA), anti-CTNNB1 antibodies (Cell Signaling Technology, Danvers, MA, USA), anti-CDH2 antibodies (Cell Signaling Technology, Danvers, MA, USA), or anti-glyceraldehyde-3-phosphate dehydrogenase (GAPDH) antibodies ((Millipore, Billerica, MA, USA). Then, the membranes were incubated with secondary anti-rabbit, anti-donkey, or anti-mouse horseradish peroxidase-conjugated antibodies (Santa Cruz Biotechnology). A gel imaging system was used to scan the protein bands using a chemiluminescent ECL reagent (Millipore). GAPDH was used as an internal reference.

## Statistical analysis

Statistical analysis was performed using SPSS ® 18.0 package for windows (IBM Corp., Armonk, NY, USA). Data are expressed as percentages. A P-value < 0.05 was considered as statistically significant. Student’s t test was used to calculate the significance of the difference between the two groups. Significant differences between multiple data sets were calculated using one-way analysis of variance (ANOVA). disease free interval survival (DFI) and progression free interval survival (PFI) were calculated using a log-rank test.

## Results

In the present study, we tried to explore the function of *LINC00313* in the Testicular Germ Cell Tumors based on a series of bioinformation analyses and *in vitro* experiments. We found *LINC00313* was higher expression in TGCT and associated significantly with prognosis of patients. Further analysis found that *LINC00313* regulated expression of EMT-related proteins and associated with immune cell infiltration. Finally, we have explored the association of LINC00313 expression and drug activity in TGCT.

## *LINC00313* CNV is associated with immune cell infiltration

**Figure 1.**
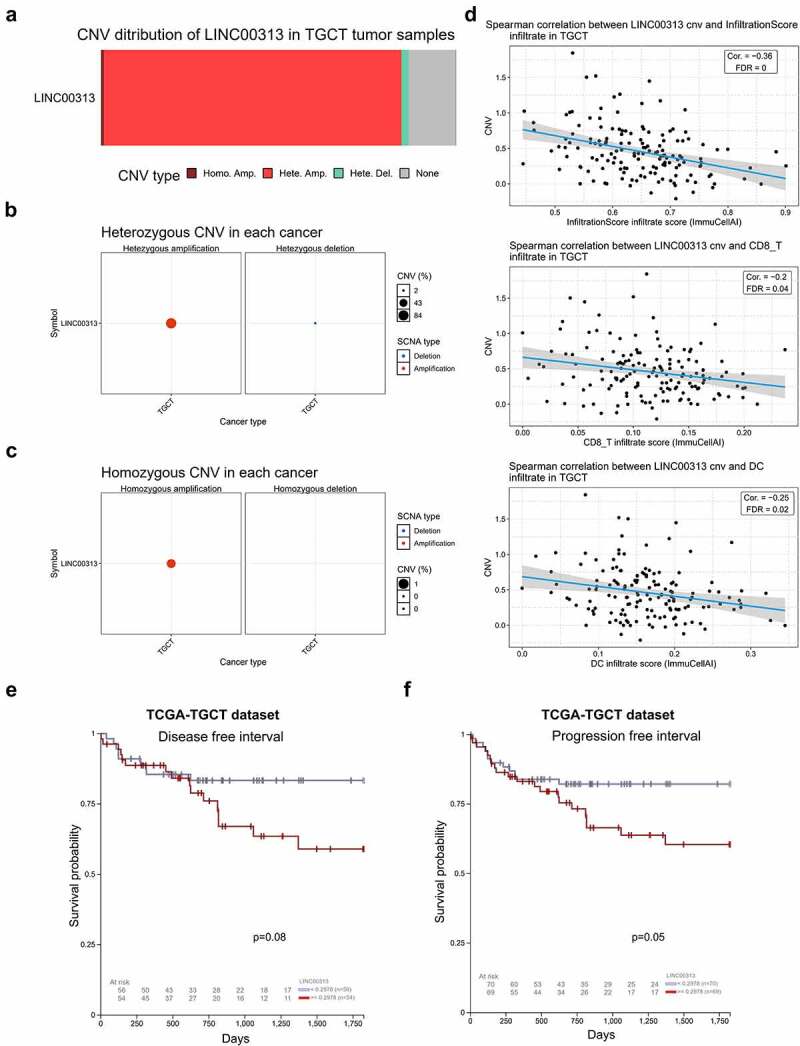
Copy number variation of *LINC00313* correlates with tumor immune invasion in TGCT. (A) Stacked plot showing the proportional distribution of copy number variation of LINC00313 in TGCT; (B) Bubble plot of heterozygous copy number variation of LINC00313 in TGCT; (C) Bubble plot of homozygosis copy number variation of LINC00313 in TGCT; (D) Correlation between LINC00313 and the immune infiltration score, CD8 + T cell infiltration, and DC cell infiltration in TGCT. (E&F) The correlation between the copy number of LINC00313 and the DFI and PFI of patients with TGCT. CNV: Copy Number Variation; TGCT: Testicular Germ Cell Tumor; Homo: Homozygous; Amp: Amplification; Del: Deletion; cor: correlation; FDR: False Discovery Rates; DC: dendritic cells; TCGA: The Cancer Genome Atlas; DFI: Disease free interval; PFI: Progression free interval.

The relationship between LINC00313 CNV and TGCT was explored. As shown in Figure 1(a,b,c) amplification was the most common CNV type of LINC00313 in TGCT, and most of the amplifications were heterozygous CNVs. Further analysis of immune cell infiltration revealed that LINC00313 CNV correlated negatively and significantly with infiltrate scores, along with CD4 + T cell and DC infiltration (Figure 1(d)). Moreover, we found that patients with a high copy number of LINC00313 had lower DFI and PFI (Figure 1(e,f)).

## Higher *LINC00313* Expression in TGCT

**Figure 2.**
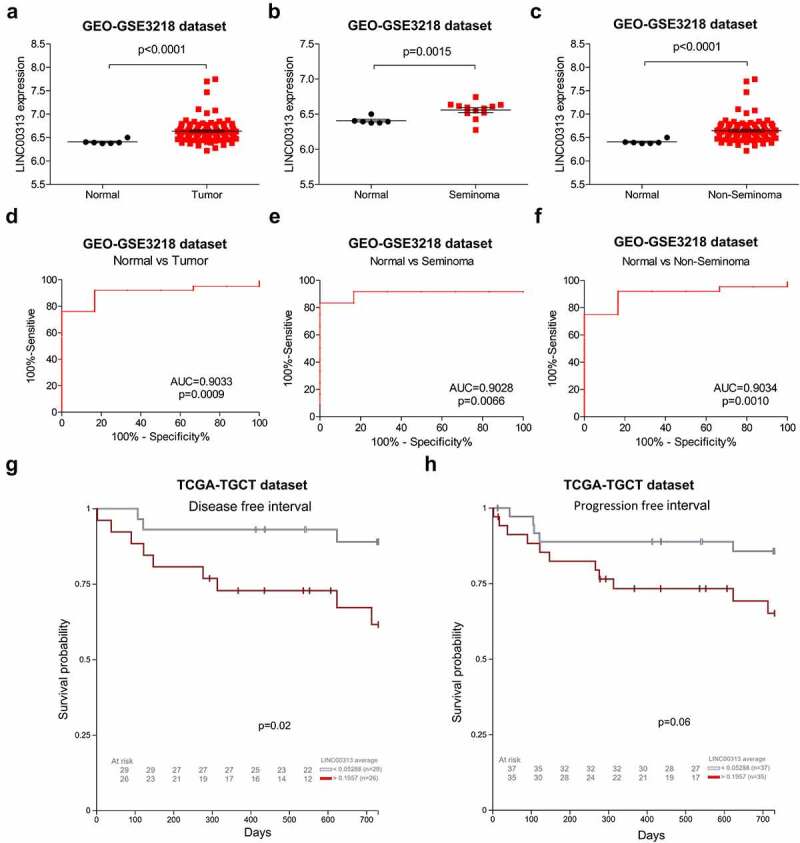
Expression of *LINC00313* correlates with the clinicopathological features of patients with TGCT. (A) GEO database analysis shows that LINC00313 was notably upregulated in TGCT. (B & C) GEO database showing that LINC00313 was notably upregulated across different subtypes of TGCT. (D, E & F) The expression of LINC00313 has high sensitivity and specificity for distinguishing normal samples from TGCT samples, seminoma samples, non-seminoma samples. (G & H) The correlation between LINC00313 and the DFI and PFI of patients with TGCT. GEO: Gene Expression Omnibus; AUC: Area Under The Curve.

To determine the expression of *LINC00313* in TGCT, TGCT data in the GEO database was used to analyze the expression of *LINC00313* statistically, which showed that *LINC00313* expression was upregulated significantly in TGCT tissues (Figure 2(a)). Meanwhile, as shown in Figure 2(b,c) *LINC00313* expression was upregulated significantly in different types of TGCT (seminoma and non- seminoma). As show in Figure 2d, e and f, the expression of *LINC00313* has high sensitivity and specificity for distinguishing normal samples from TGCT samples, seminoma samples, non- seminoma samples. Moreover, *LINC00313* expression was also associated significantly with DFI and PFI survival in patients with TGCTs (Figure 2 g, h). Thus, *LINC00313* could be used as an independent diagnosis and prognostic indicator for patients with TGCT.

## Silencing of *LINC00313* inhibited the migration and invasion of TGCT cells

**Figure 3.**
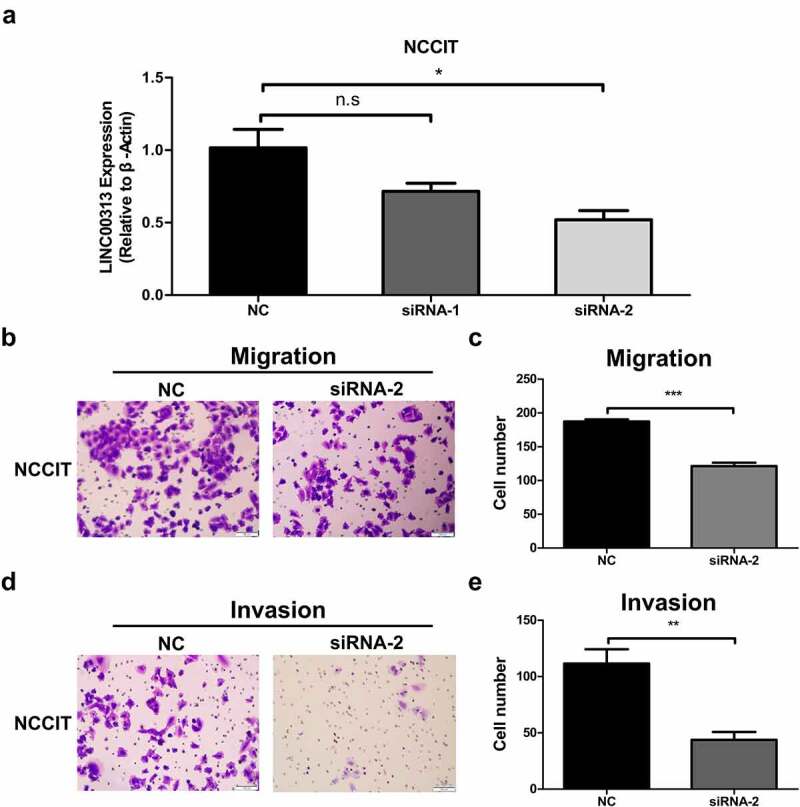
The impact of *LINC00313* on the migratory and invasive properties of TGCT. (A) The silencing efficiency of LINC00313 siRNA on the TGCA cell line was assessed using qRT-PCR. (B&C) Transwell cell migration assays enabled an assessment of how LINC00313 silencing affected TGCT cell migration (D&E) Transwell cell invasion assays enabled an assessment of how LINC00313 silencing affected TGCT cell invasion. NC: Negative Control; siRNA: small interfering RNA.

To determine whether *LINC00313* can promote the migration and invasion of TGCT, we selected NCCIT cells for further *in vitro* experiments. The expression levels of *LINC00313* in NCCIT was determined using qRT-PCR to verify the effects of the silencing of *LINC00313* (Figure 3a). The migration and invasion abilities of TGCT cells was investigated using the Transwell assay(Figure 3b, c, d and e). *LINC00313* silencing decreased the number of invaded and migrated cells significantly. This indicates that *LINC00313* plays an important role in the migration and invasion of TGCT, which warrants further investigation.

## LINC00313 regulates expression of EMT-related proteins

**Figure 4.**
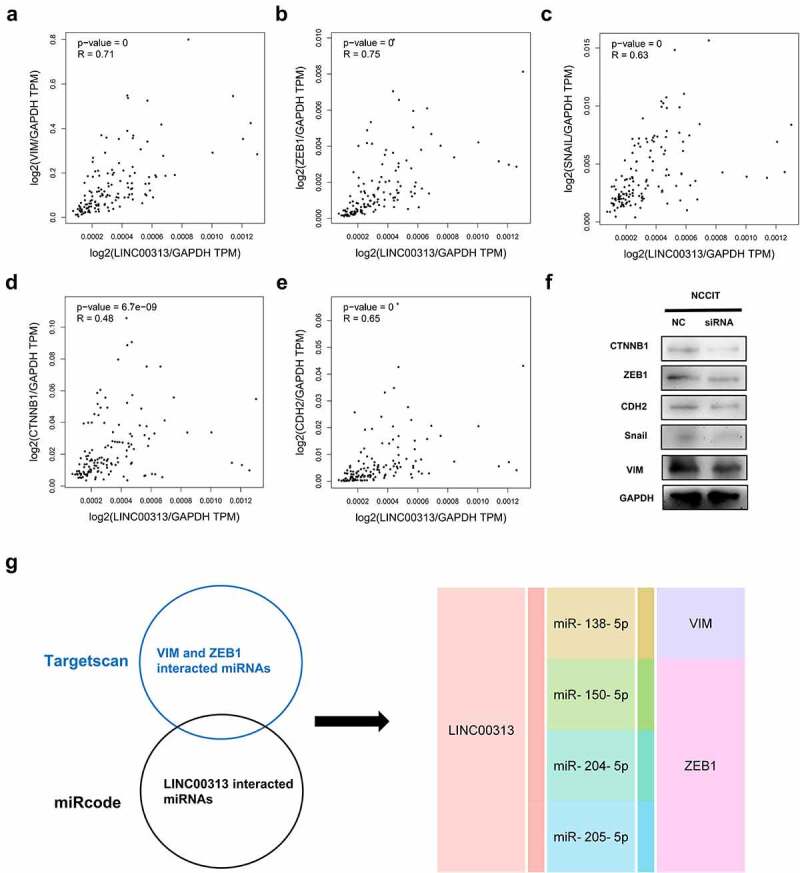
Effects of silencing of *LINC00313* on EMT-related proteins were investigated using Western blotting experiments. (A–E) The association between LINC00313 and EMT markers were analyzed with the GEPIA database (F) After silencing of LINC00313, the levels of VIM, ZEB1, SNAIL, CTNNB1, and CDH2 were downregulated significantly. (G) LINC00313-miRNA-gene interaction network. TPM: Transcripts Per Kilobase Million.

The EMT signaling pathway has been implicated in several types of cancers. Notably, *LINC00313* has previously been found to be linked to the EMT process in thyroid cancer cells [19]. Based on this, we analyzed the relationship between *LINC00313* and EMT markers (VIM, ZEB1, SNAIL, CTNNB1, and CDH2) using the GEPIA database and found that *LINC00313* expression correlated significantly and positively with these EMT markers (Figure 4a–e). Therefore, we speculated that *LINC00313* might be involved in process of TGCT by regulating EMT signals. Therefore the expression of EMT-related proteins after silencing of *LINC00313* was assessed using western blotting. The levels of VIM, ZEB1, SNAIL, CTNNB1, and CDH2 proteins were downregulated significantly (Figure 4f), suggesting that the *LINC00313*-mediated EMT might be an important mechanism involved in TGCT migration and invasion. Then, we have analyzed interacted miRNAs associated with the lncRNA and genes, and drawn a lncRNA-miRNA-gene interaction network (Figure 4g). *LINC00313* might mediate EMT through miR-138-5p, miR-150-5p, miR-204-5p and miR-205-5p.

**Figure 5.**
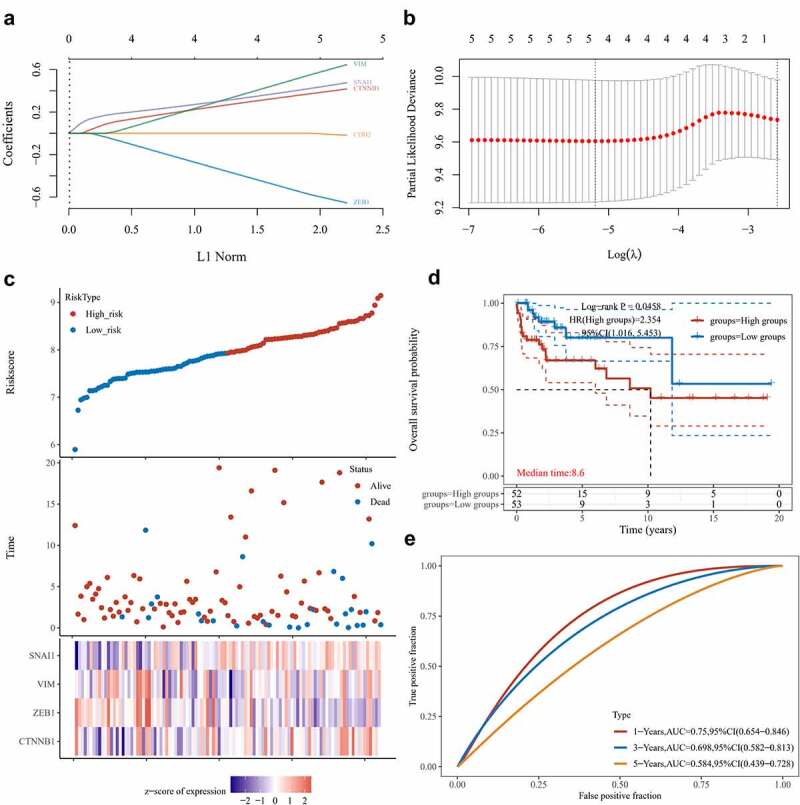
Establishing a prognostic EMT-related gene risk signature for TGCT patients in the TCGA TGCT cohort. (A)LASSO Cox regression of the 5 EMT-related genes regulated by LINC00313. (B) Screening of the parameter in the LASSO Cox regression. (C) After dividing patients into high- and low-risk groups, the risk distribution, survival, and expression of 4 related genes for each patient was displayed. (D) Significant differences of DFS were observed between high-and low-risk TCGA TGCT patients (E) ROC curves exhibits the predictive sensitivity and specificity of the risk score at 1, 3, and 5 years. HR: hazard ratio.

We proceeded to build a risk model through Lasso regression with reference to the EMT-related genes regulated by LINC00313 (Figure 5a). The combination of 4 genes out of 5 input genes yielded the highest C-index (Figure 5b), Risk score = (0.3675)*CTNNB1+(−0.5661)*ZEB1+(0.5392)*VIM+(0.4163)*SNAI1, lambda.min = 0.0056. Based on our model, we stratified patients according to low- and high-risk groups (Figure 5c). We noted that as the risk score increased, the patient’s mortality rate also gradually increased (Figure 5c). Patients in the high-risk group possessed a poorer DFS (Figure 5d). Finally, we plotted a time-dependent ROC curve and found that the AUCs at 1, 3, and 5 years were 0.75, 0.698, and 0.584, respectively(Figure 5e).

## *LINC00313* is associated with immune cell infiltration

**Figure 6.**
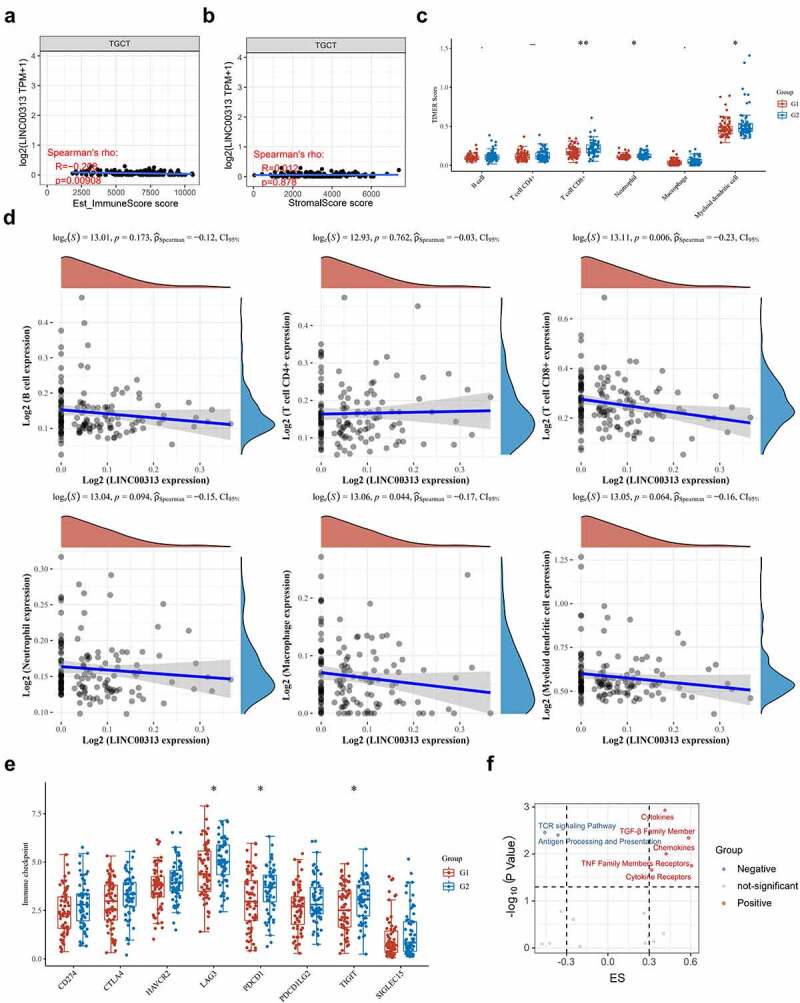
Relationship between *LINC00313* and immune cells and immune pathways in the TGCT immune microenvironment. (A) Correlation between LINC00313 and immune score. (B) Correlation between LINC00313 and stromal score. (C) Difference in immune cell score between high and low expression of LINC00313 in TGCT patients. (D) Correlation between LINC00313 and immune cell enrichment scores. (E) Differential expression of immune checkpoint genes between high and low expression of LINC00313 in TGCT patients. (F) Correlation between LINC00313 and the enrichment scores of various immune-related pathways. G1: LINC00313 high group; G2: LINC00313 low group. Est: Estimation; ES: Enrichment score.

The relationship between the TGCT immune and stromal scores and *LINC00313* was explored using the TIMER software. Our analysis found that *LINC00313* expression correlated significantly and negatively with the estimated score and the immune score but not with the stromal score (Figure 6 and b). Meanwhile, the quantities of CD8 + T cells, neutrophils, and dendritic cells were significantly lower in TGCT samples with high LINC00313 expression (Figure 6c). Furthermore, Spearman correlation analysis revealed that CD8 + T cells and DCs infiltration negatively correlated with *LINC00313* expression (Figure 6d). The lower the expression of *LINC00313*, the higher the degree of CD8 + T cells and DCs tumor infiltration. Additionally, we also noted a significantly suppressed expression of immune checkpoint genes LAG3, PDCD1, and TIGIT in TGCT samples with high *LINC00313* expression (Figure 6e). Candidate immune signaling pathways influenced by *LINC00313* were investigated using the ImmuLnc tool. We found that levels of cytokines, transforming growth factor-beta (TGF-β) family members, chemokines, tumor necrosis factor (TNF) family members receptors, and cytokine receptors were raised when *LINC00313* was overexpressed, while the T cell receptor (TCR) signaling pathway and antigen processing and presentation were reduced after *LINC00313* overexpression (Figure 6f). Based on the above findings, we hypothesize that the TGCT tumor immune response and immune microenvironment were likely modulated by *LINC00313*.

## Potential drug targeting *LINC00313* in TGCTs

**Figure 7.**
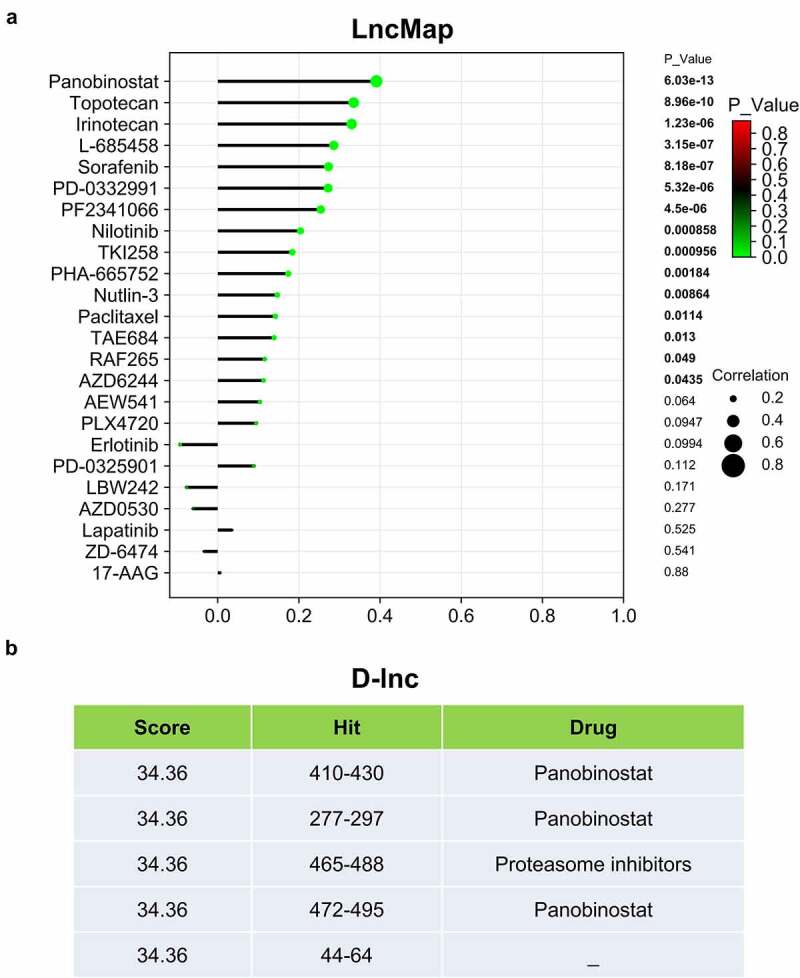
*LINC00313* related drug screening. (A) LncMap analysis of the correlation between *LINC00313* expression and the IC50 of various drugs. (B) D-lnc online tool predicts the score and binding site of drugs that may interact with *LINC00313*. Hit represents the position where the drug acts on *LINC00313.*

Our previous results indicated that *LINC00313* potentially affects the migration and invasion of TGCTs, thus highlighting *LINC00313* as a novel therapeutic target. Therefore, we explored the association of *LINC00313* expression and drug activity in TGCTs. We used the LncMap tool to calculate the Spearman correlation coefficient between *LINC00313* expression levels and the IC50 values of drugs. We observed that the *LINC00313* expression was influenced by 15 drugs, among which panobinostat was found to be the best candidate drug to target *LINC0031*3 in TGCT (Figure 7a). In addition, we also performed the same analysis using another prediction tool D-lnc. Interestingly, panobinostat was also found to be a drug that was highly compatible and had multiple potential binding sites (Figure 7b).

## Discussion

The most frequent cancer in the young male population is TGCT. Radical orchiectomy is typically the first treatment for all testicular cancers, which has a high cure rate [20]. However, in the patients with refractory disease, there are few alternative treatments. Meanwhile, although therapeutic approaches have improved, approximately 25% of the patients had relapse [21]. Hence, the metastasis and recurrence of TGCT are one of the most intractable problems in the clinical setting. Previous studies have confirmed that AKT3 expression was higher in TGCT patients and might be a potential therapeutic target and a novel molecular marker of TGCT [22]. Wu etc. have reported that Toll-like receptor 2 (TLR2) might affect the prognosis of TGCT, as well as be an indicator of immune function in the tumor microenvironment [23]. However, the roles of lncRNAs in TGCT have been less studied before. In recent years, research on lncRNAs has received increased attention and has become a hotspot of tumor research [24]. LncRNAs plays a crucial role in tumorigenesis, invasion, metastasis, and recurrence. There was a study reported that H19, a Long Non-coding RNA, played an oncogenic role in pan-cancer including TGCT and showed that miRNA-mediated lncRNA-TF-gene co-regulation is complicated yet important in cancers. And suggested that co-regulation-based approach is promising to identify TFs or genes related to cancer [25]. Here, we especially report the role of *LINC00313* in TGCT, while also referring to the valuable experience of previous studies.

In previous studies, *LINC00313* was shown to be upregulated and correlated with poor prognosis in some cancers. For example, *LINC00313* was reported to be highly expressed in lung cancer and to indicate shorter overall survival (OS) [26]. High expression of *LINC00313* was associated with shorter OS in osteosarcoma [27]. To the best of our knowledge, the expression and role of *LINC00313* in TGCT has not been reported. In the present study, we performed data mining using public databases to show that *LINC00313* expression was upregulated significantly in TGCT tissue and that there was significantly association between DFI survival and *LINC00313* expression. This is similar to the findings of another research team in osteosarcoma [27] and cervical carcinoma [28], who found that patients with high *LINC00313* expression had poorer overall and disease-free survival. Moreover, the expression of *LINC00313* has a good diagnostic value for TGCT. These results indicate that *LINC00313* may be a potential diagnostic and prognostic marker for TGCT.

We also found significant associations between *LINC00313* and tumorigenesis and metastasis. *LINC00313* knockdown suppressed cell migration and invasion in the TGCT cell line. These data support the view that *LINC00313* plays a very important role in the migration and invasion of TGCT cells. Previous studies demonstrated that *LINC00313* participates in the progression of various tumors [29,30], which was consistent with our findings. The mechanism of *LINC00313* dysregulation in TGCT requires further research, although our results showed that *LINC00313* promote the migration and invasion abilities of TGCT cell lines. To our knowledge, our study is the first to characterize the functional role of *LINC00313* in TGCTs.

EMT is critical for local invasion and cell dissemination, which in cancer, are associated with tumor initiation and progression, stemness, survival, and resistance to therapy [31]. In our study, the western blotting showed that *LINC00313* might exert its tumor-promotive role by modulating EMT signaling, which was consistent with the results of cell invasion and migration. EMT plays an important role in carcinogenesis, especial for local invasion and cell dissemination. Previous studies have also indicated that *LINC00313* accelerated the progression, migration, and EMT of cancer cells, including cervical carcinoma [28] and papillary thyroid carcinoma [19,30]. However, whether *LINC00313* participates in the EMT process in TGCTs remains to be further confirmed.

The tumor microenvironment (TME) has been widely implicated in tumorigenesis because it harbors tumor cells that interact with surrounding cells through the circulatory and lymphatic systems to influence the development and progression of cancer [32]. Infiltrating inflammatory cells in the TME play an important role in determining tumor survival and patient prognosis [33]. In recent years, the study of the involvement of lncRNAs in immune regulation has become a research hot spot. In the present study, we found that there was a close relationship between *LINC00313* and immunity in tumor tissues. *LINC00313* expression correlated significantly and negatively with estimate score and immune score and was correlated significantly and negatively with CD8 + T cells and dendritic cells in the TME. Meanwhile, *LINC00313* CNV also correlated significantly and negatively with CD4 + T cells and dendritic cells. Our results suggested that *LINC00313* might be involved in the tumor immune process and the expression level of *LINC00313* might serve as a predictor of the response to tumor immunotherapy. However, this hypothesis should be confirmed by further *in vivo* experiments in animals. This is also a limitation of the study.

The cost of developing drugs has always been high; therefore, most potential drugs are tested through drug activity screening *in vitro* with lower cost and shorter time. Based on the drug activity screen data, we revealed the associations among LINC00313 and cancer drugs. Panobinostat was identified as the best possible candidate drugs to target *LINC00313* in TGCTs. However, the evaluation of lncRNAs for drug therapy is still in its early stages [34].

## Conclusion

In summary, we report the role of *LINC00313* in TGCTs. We identified that *LINC00313* performs important pro-migration and invasion functions in TGCT pathogenesis. Our results indicated that *LINC00313* might be a diagnostic, prognostic, immune marker and therapeutic target for TGCTs, and will facilitate accurate and effective treatment of TGCTs.

## Abbreviations

TGCT: Testicular germ cell tumor;
lncRNAs: Long noncoding RNAs
GEO: Gene Expression Omnibus
CNV: copy number variation
TCGA: The Cancer Genome Atlas
PGCs: primordial germ cells
ncRNAs: non-coding RNAs
miRNAs: microRNAs
piRNAs: piwi-interacting RNAs
GSCA: Gene Set Cancer Analysis
DCs: dendritic cells
GEPIA: Gene Expression Profiling Interactive Analysis
GTEx: Genotype-Tissue Expression
EMT: epithelial-mesenchymal transition
RPMI: Roswell Park Memorial Institute
FBS: fetal bovine serum
siRNA: small interfering RNA
qRT-PCR: Quantitative real-time reverse transcription PCR
RIPA: radioimmunoprecipitation assay
SDS-PAGE: sodium dodecyl sulfate-polyacrylamide gel electrophoresis
PVDF: polyvinylidene difluoride
ANOVA: one-way analysis of variance
DFI: disease free interval survival
PFI: progression free interval survival
TGF-β: transforming growth factor beta
TNF: tumor necrosis factor
TCR: T cell receptor
TME: The tumor microenvironment
OS: overall survival.
